# Genetic Biomarkers on Age-Related Cognitive Decline

**DOI:** 10.3389/fpsyt.2017.00247

**Published:** 2017-11-21

**Authors:** Chieh-Hsin Lin, Eugene Lin, Hsien-Yuan Lane

**Affiliations:** ^1^Graduate Institute of Biomedical Sciences, China Medical University, Taichung, Taiwan; ^2^Department of Psychiatry, Kaohsiung Chang Gung Memorial Hospital, Chang Gung University College of Medicine, Kaohsiung, Taiwan; ^3^Center for General Education, Cheng Shiu University, Kaohsiung, Taiwan; ^4^Department of Electrical Engineering, University of Washington, Seattle, WA, United States; ^5^TickleFish Systems Corporation, Seattle, WA, United States; ^6^Department of Psychiatry, Brain Disease Research Center, China Medical University Hospital, Taichung, Taiwan

**Keywords:** Alzheimer’s diseases, biomarker, age-related cognitive decline, cognitive aging, gene–gene interactions, neurodegeneration, single-nucleotide polymorphisms, SNP–SNP interactions

## Abstract

With ever-increasing elder populations, age-related cognitive decline, which is characterized as a gradual decline in cognitive capacity in the aging process, has turned out to be a mammoth public health concern. Since genetic information has become increasingly important to explore the biological mechanisms of cognitive decline, the search for genetic biomarkers of cognitive aging has received much attention. There is growing evidence that single-nucleotide polymorphisms (SNPs) within the *ADAMTS9, BDNF, CASS4, COMT, CR1, DNMT3A, DTNBP1, REST, SRR, TOMM40*, circadian clock, and Alzheimer’s diseases-associated genes may contribute to susceptibility to cognitive aging. In this review, we first illustrated evidence of the genetic contribution to disease susceptibility to age-related cognitive decline in recent studies ranging from approaches of candidate genes to genome-wide association studies. We then surveyed a variety of association studies regarding age-related cognitive decline with consideration of gene–gene and gene–environment interactions. Finally, we highlighted their limitations and future directions. In light of advances in precision medicine and multi-omics technologies, future research in genomic medicine promises to lead to innovative ideas that are relevant to disease prevention and novel drugs for cognitive aging.

## Introduction

Generally speaking, age-related cognitive decline, or cognitive aging, is recognized as a gradual and enduring process of transition in cognitive capacity with increasing age (1). Although cognitive decline is common in old age, the relationship between aging and neurodegenerative disease such as dementia remains unclear. Whereas aging is a well-known risk factor for dementia, dementia is not an inevitable consequence of the process of aging. The concept and underlying mechanisms of normal aging and pathological aging might be different. It should be noted that cognitive aging may raise the likelihood of many age-associated diseases and neurodegenerative disorders, such as mild cognitive impairment (MCI), Alzheimer’s diseases (AD), Parkinson’s disease, and other dementias, due to the fact that prior research work has projected that rates of age-associated diseases and neurodegenerative disorders gain rapidly with advancing age (2). While ever-increasing elder populations exist in both developed and developing countries, the pervasiveness of age-associated diseases and neurodegenerative disorders has become a huge public health concern owing to high social and economic burdens (3). What is more, cognitive aging processes implicate multiple complex pathogeneses including genetic and environmental factors (4). In this light, the identification of genetic biomarkers has become an important area of research that aims to preclude the advancement of cognitive aging and to grasp the biology of cognitive aging in an increasingly aging society (5). It is noteworthy that untangling genetic biomarkers for cognitive aging has been at the center of major investigations in the field of precision medicine, and the relevant biomarkers for AD are generally utilized in cognitive aging research as well because of the increased risk for AD in the elderly individuals (6).

More recent research in genome-wide association studies (GWAS) has implicated that single-nucleotide polymorphisms (SNPs) among 11 genes appear to affect the risk of AD, encompassing the *PICALM, MS4A6E, MS4A4E, MS4A4A, EPHA1, CR1, CLU, CD33, CD2AP, BIN1, APOE*, and *ABCA7* gene (7–11). Consequently, a meta-analysis of GWAS studies (*n* = 74,046) identified 14 risk genes in AD, encompassing the *ZCWPW1, SORL1, SLC24A4, RIN3, PTK2B, NME8, MEF2C, INPP5D, HLA-DRB4, HLA-DRB1, FERMT2, DSG2, CELF1*, and *CASS4* genes (12). The succeeding whole-exome sequencing analysis further tracked down the *PLD3* gene to likely be a risk gene for AD (13). Moreover, it has been found that cognitive decline was linked with the *CR1* rs6656401 SNP by using the established AD-associated genes (14). Additionally, recent epistasis studies suggested that the *CLU*-*MS4A4E* (15, 16) and *CD33*-*MS4A4E* (16) gene–gene interactions might have a considerable influence on the susceptibility of AD. By utilizing the known AD-associated genes, it has also been demonstrated that cognitive decline was related with the *ABCA7* rs3764650 and *CD33* rs3865444 SNPs in the elderly Caucasian women (17).

In this review, we first surveyed some genetic biomarkers that were linked with age-related cognitive decline in several recent association studies (Table 1). Furthermore, we assessed some potential gene–gene and gene–environment interactions on age-related cognitive decline. This review does not intend to comprehensively survey all literature. We mainly focused on the most recent developments for biomarker research in cognitive aging. Finally, the limitations and future perspectives associated with cognitive aging in terms of genetic biomarkers were summarized. Future replication studies in larger samples with longitudinal follow-up are required to confirm the findings of the biomarkers for cognitive aging discovered in the association studies.

**Table 1:** Relevant studies in genetic biomarkers on age-related cognitive decline.

Gene	Study	Ethnic group	Results
*ADAMTS9*	Lin et al. (56)	Taiwanese (mean age: 64.2)	*ADAMTS9* (including rs9831846, rs4317088, rs9985304, and rs73832338) were linked with cognitive aging

*APOE*	De Jager et al. (30)	Various populations (mean age: 72.0~80.8)	*APOE* was genome-wide significantly correlated with cognitive aging for normal aging

*BDNF*	Laing et al. (34)	German (mean age: 72.7)	*BDNF* altered cognitive aging in healthy subjects for normal aging

*CASS4*	Lin et al. (18)	Taiwanese (mean age: 64.2)	*CASS4* rs911159 persisted significant for cognitive aging after Bonferroni correction for normal aging

*COMT*	Liu et al. (36); Papenberg et al. (37)	Taiwanese (mean age: 78.7) German (mean age: 64.9~65.3)	*COMT* Val158Met contributed to individual differences in cognitive aging

*CR1*	Chibnik et al. (14)	non-Hispanic white (mean age: 75.5~84.4)	*CR1* was significantly associated with cognitive aging for normal and pathologic aging

*DNMT3A*	Chouliaras (78)	Dutch (mean age: NA)	*DNMT3A* rs11887120 was associated with cognitive decline for normal aging

*REST*	Lin et al. (67)	Taiwanese (mean age: 64.2)	*REST* rs1277306 was linked with cognitive aging for normal aging

*TOMM40*	Davies et al. (86)	Various populations (mean age: 64.6~79.1)	*TOMM40* rs2075650 was significantly associated with cognitive aging for normal aging

Circadian clock genes	Lin et al. (42)	Taiwanese (mean age: 64.2)	*RORA* rs13329238, *NPAS2* rs17655330, *CLOCK* rs3749473, and *RORB* rs10781247 individually and interactively altered cognitive aging for normal aging

## Recent Association Studies

### AD-Associated Genes

As mentioned previously in the Section “Introduction,” it has been revealed that AD risk is linked with *ZCWPW1, SORL1, SLC24A4, RIN3, PTK2B, PLD3, PICALM, NME8, MS4A6E, MS4A4E, MS4A4A, MEF2C, INPP5D, HLA-DRB4, HLA-DRB1, FERMT2, EPHA1, DSG2, CR1, CLU, CELF1, CD33, CD2AP, CASS4, BIN1, APOE*, and *ABCA7 in* GWAS and meta-analyses (7–12). To recognize probable genes implicated in the regulation of age-related cognitive decline, a recent association study has analyzed whether SNPs within these 27 AD-associated genes are linked with cognitive aging as well as via complex gene–gene and gene–environment interactions in a cohort of older Taiwanese adults (*n* = 634) aged over 60 years (mean age: 64.2 years) from the Taiwan Biobank (18). In order to weigh cognitive functions, the mini-mental state examination (MMSE) method was administered for all participants (18). Lin et al. tested 588 SNPs, but only the *CASS4-*rs911159 SNP persisted significant for cognitive aging after Bonferroni correction (18). In addition, their analysis results suggested an association with 6 more SNPs in the AD-related genes, encompassing the *SLC24A4-*rs67063100, *RIN3-*rs1885747, *PLD3-*rs11672825, *MEF2C-*rs9293506, *FERMT2-*rs4901317, and *EPHA1-*rs10952552 SNPs (18). Lin et al. also displayed the gene–gene interactions among the *SLC24A4-*rs67063100, *MEF2C-*rs9293506, *FERMT2-*rs4901317, *EPHA-*rs10952552, and *CASS4-*rs911159 SNPs on cognitive aging by using the generalized multifactor dimensionality reduction (GMDR) approach (18). Furthermore, they disclosed the gene–environment interactions of the *MEF2C-*rs9293506 and *SLC24A4-*rs67063100 SNP with environmental factors including social support, physical activity, smoking status, and alcohol consumption on cognitive aging by using the GMDR approach (18).

Consistent with the findings by Lin et al. (18), two preceding GWAS studies (8, 10) have reported that the *EPHA1* rs11767557 SNP may affect the vulnerability to AD. In addition, the *FERMT2* rs17125944, *MEF2C* rs190982, and *SLC24A4* rs10498633 SNPs were prone to AD in a meta-analysis study (12). Moreover, a whole-exome sequencing study pinpointed a rare rs145999145 (Val232Met) variant in the *PLD3* gene was liable to AD (13). The *PLD3* gene may play a central role in dealing with amyloid-beta precursor protein (13).

The CASS4 protein is proposed to better characterize the functions of cell growing, spreading, adhesion, and other activities (19). Moreover, it was speculated that *CASS4* plays a vital part in the hallmarks of AD such as the amyloid precursor protein (APP) and Tau protein (20). More recent studies also indicated an association between AD and the *CASS4* rs7274581, rs6024870, and rs16979934 SNPs (12, 21–23). In contrast, a replication study indicated that the *CASS4* rs7274581 SNP did not affect the risk of AD in a Spanish population (24).

Gene–gene interaction study inferred that *SLC24A4, MEF2C, FERMT2, EPHA1*, and *CASS4* synergistically raised the propensity of cognitive aging by using the GMDR approach (18). It was further speculated that these five genes are comprised in the relevant pathology and pathways (18). *EPHA1* is implicated in regulating neurodevelopment (20). *FERMT2* contributes to Tau neurotoxicity and cell adhesion (25). *MEF2C* may influence hippocampal synaptic connectivity and thereby mediate hippocampal-dependent memory and learning (26). *SLC24A4*, in the vicinity of the *RIN3* gene, is linked with neurodevelopment (27) as well as Tau pathology and APP (28).

### *APOE* 

The *APOE* gene, located on chromosome 19q13.32, encodes a major protein which is crucial for the regular catabolism of triglyceride-rich lipoprotein constituents (29). Conducting a GWAS study with the longitudinal cognitive testing data such as memory and perceptual speed, De Jager et al. discovered that *APOE* was genome-wide significantly correlated with age-related cognitive decline (mean age: 72.0~80.8 years) (30). A meta-analysis of 77 studies (*n* = 40,942) also suggested that carriers of the *APOE* ɛ4 allele, which were linked with late-onset AD, performed worse on several domains of cognitive functions including overall global cognitive ability, episodic memory, and executive functioning (31). Further, the *APOE* rs405509 and *APOE* rs440446 SNPs were more likely to develop non-pathological cognitive aging, independent of *APOE* major isoforms, in a Finnish population (32). In contrast, a replication study indicated that the *APOE* gene did not affect the risk of age-related cognitive decline in older Taiwanese adults (18).

### *BDNF* 

Another potential candidate gene such as *BDNF* was reported to be implicated in age-related cognitive decline among the elderly (33). The *BDNF* gene, encoding proteins of the nerve growth factor family, was demonstrated to alter cognitive deficits in healthy subjects (mean age: 72.7 years) for normal aging by using the mean Z-scores based on three cognitive domains including motor function, memory, and perceptual speed (34). Ward et al. also disclosed an interaction between *APOE* and *BDNF* that predicted a cognitive effect in healthy older adults (35).

### *COMT* 

The *COMT* gene, located on chromosome 22q11.21, is essential in the metabolic degradation of prefrontal dopamine (36). A functional *COMT* Val158Met polymorphism has been shown to contribute to individual differences in cognitive aging in Taiwanese (mean age: 78.7 years) and German (mean age: 64.9~65.3 years) populations using MMSE scores (36, 37). Based on a 4-year longitudinal study, Dixon et al. also revealed that the *APOE* and *COMT* genes are complementary biomarkers in normal cognitive aging and early MCI for older adults (38).

### *CR1* 

The *CR1* gene encodes a protein that plays a role in cellular binding and immune complexes (14). A replication study reported that the rs6656401 SNP in the *CR1* gene, which is one of the susceptibility loci for AD, was significantly associated with cognitive deficits (mean age: 75.5~84.4 years) for normal aging (in terms of global cognitive decline) as well as for pathological aging (in terms of global AD pathology) using the cognitive testing data such as memory and global cognition (14). A subsequent replication study also indicated that the rs4844609 SNP in the *CR1* gene modulates episodic memory decline and an interaction between *APOE* and *CR1* influences cognitive decline for normal aging and for pathological aging (39). In contrast, a replication study indicated that the *CR1* gene did not affect the risk of age-related cognitive decline in older Taiwanese adults (18).

### Circadian Clock Genes

Circadian rhythms are instinctively recurring cycles that determine the timing of biological events such as energy metabolism, hormone release, and sleep–wake cycles (40). Additionally, dysregulation of circadian rhythms is characteristic of the natural process of ongoing aging and cognitive decline (40). The circadian rhythms are maintained and triggered by a composition of core circadian clock genes, which can be classified as vital genes on provoking circadian rhythms in specific cells (41).

Circadian clock genes, encompassing *RORB, RORA, PER3, PER2, PER1, NR1D1, NPAS2, CRY2, CRY1, CLOCK*, and *ARNT*, may be also involved in cognitive impairment (40, 41). To find out potential genes implicated in the regulation of age-related cognitive decline, a recent study analyzed whether the 11 aforementioned core circadian clock genes as well as complex gene–gene and gene–environment interactions contributed to cognitive aging in more than 634 elderly individuals (mean age: 64.2 years) in Taiwan (42). As a result, four SNPs, including the *RORA*-rs13329238, *NPAS2-*rs17655330, *CLOCK-*rs3749473, and *RORB*-rs10781247 SNPs individually and interactively alters the hazard of cognitive deficits in terms of MMSE scores for normal aging (42). Finally, environmental factors such as smoking status, alcohol consumption, social support, and physical activity also interacted with these SNPs in regulating the liability of age-related cognitive decline (42).

*CLOCK* encodes a protein which creates the ARNTL/CLOCK heterodimeric protein with ARNTL (43). In addition, rs1554483, 3111, and rs4580704 *CLOCK* SNPs influenced the tendency to AD in Chinese subjects (44–46). However, these results were not confirmed in different populations (42).

The RORA and RORB proteins create a family of nuclear hormone receptors (47). These two genes were reported to have a key part in a wide variety of regulations; for instance, cellular metabolism, circadian rhythm, embryonic development, immunity, and inflammatory responses (48, 49).

It should be noted that *NPAS2* encodes transcription factors which are a component belonging to the basic helix-loop-helix family, and the ARNTL/NPAS2 and ARNTL/CLOCK heterodimeric proteins can bind to chromatin, bringing about the upregulation in gene expression for *PER1, PER2, PER3, CRY1*, and *CRY2* (50). In this manner, products of these *CRY1, CRY2, PER1, PER2*, and *PER3* genes build a complex that inhibits the action of the ARNTL-containing complexes and in turn forms a negative feedback loop (50).

### Insulin Resistance-Associated Genes

To identify possible genes implicated in the process of age-related cognitive decline, a recent association study of the Taiwan Biobank has hypothesized that SNPs in insulin resistance-associated genes, including the *PPARG, GCKR*, and *ADAMTS9* genes, might be associated with cognitive aging individually or collectively in a cohort of Taiwanese individuals (*n* = 547; age > 60 years; mean age: 64.1 years) (51). The results indicated that 4 SNPs (including rs9831846, rs4317088, rs9985304, and rs73832338) within *ADAMTS9* were linked with MMSE scores (that is, cognitive aging) after performing Bonferroni correction (51). In addition, Lin et al. identified a SNP–SNP interaction between *ADAMTS9*-rs76346246 and *ADAMTS9-*rs9985304 that may influence cognitive aging by using the GMDR approach (51).

*ADAMTS9* is demonstrated in the regulation of a wide variety of processes, such as the control of organ shape during growth, the inhibition of angiogenesis, and the cleavage of proteoglycans (51). Since one of the risk factors for cognitive aging and AD is insulin resistance, insulin resistance-related genes might be linked with cognitive aging (52, 53). Insulin abnormalities raise the uncertainty for neurodegenerative disorders including MCI, AD, and cognitive aging (54, 55). Insulin is also a fundamental factor for normal brain functioning. It should be noted that *ADAMTS9* is associated with insulin resistance, insulin sensitivity, and type 2 diabetes (56, 57). There is growing evidence that the ADAMTS-9 protein might participate in the processes of brain disease states including spinal cord injury, ischemic stroke, and transient middle cerebral artery occlusion in animal studies (58–60). In accordance, a GWAS study has identified the *ADAMTS9* rs6795735 SNP as a candidate biomarker for age-related macular degeneration, which commonly occurs in elder adults (61).

### RE1-Silencing Transcription Factor (*REST*) Gene

A growing body of evidence suggests that *REST* may be involved with AD and cognitive aging (62). A recent replication study has assessed whether *REST* SNPs are associated with cognitive aging as well as via SNP–SNP interactions for normal aging in elder Taiwanese subjects (*n* = 634; mean age: 64.2 years) (62). Their analysis results demonstrated that *REST*-rs1277306 was linked with cognitive aging, which was measured by MMSE scores (62). This prediction is further supported by evidence that the association remained significant for individuals without *APOE* ε4 allele after Bonferroni correction (62). On the other hand, the *REST* rs1277306 SNP was not a predicting factor for cognitive aging among individuals with at least one *APOE* ε4 allele (62). In addition, Lin et al. tracked down an SNP–SNP interaction between the *REST* rs1713985 and *REST* rs1277306 SNPs on cognitive aging by using the GMDR. The REST protein is indicated in the modulation of synaptic plasticity, ion channels, vesicular transport, axonal growth, and neuronal differentiation (63, 64). In addition, *REST* is associated with amyloid β-protein toxicity, protection from oxidative stress, AD pathology, MCI, brain aging, and slow hippocampal loss (65–67). Additionally, *REST* might act as both a primary protector against neurodegeneration and an essential repressor for normal neurogenesis (65–67). Although *APOE* is well-established regarding its major role in cognitive decline in elder adults (68, 69), the biologically synergistic effects between the *APOE* and *REST* genes on cognitive aging are still unknown. It was speculated that *APOE* and *REST* might involve in a comparable pathway relevant to cognitive aging (70). Consistent with the findings by Lin et al. (62), several other studies pinpointed an interaction of *APOE* with *PSEN2* (71), *PSEN1* (72), *PICALM* (70), and *APP* (71, 73) by using patient stratification based on *APOE* ε4 status.

### DNA Methylation

Recent studies indicate that DNA methylation, one of main epigenetic mechanisms, plays a crucial role in cognitive aging (74, 75). DNA methylation involves the inclusion of a methyl group to the DNA molecule, especially when a cytosine is followed by a guanine (76). DNA methylation is regularly associated with reduced transcriptional activity and is triggered by a family of DNA methyl-transferase proteins (77). Using repeated measures of composite scores for annual cognitive testing, Chouliaras et al. investigated associations among common SNPs in genes modulating DNA methylation and cognitive aging. They found that the rs11887120 SNP in the *DNMT3A* gene was associated with annual decline in cognitive deficits for normal aging in a Dutch population (78). In contrast, this finding was not replicated in German subjects (79). *DNMT3A*, encoding a DNA methyltransferase, is located in the cytoplasm and nucleus in *de novo* methylation (80).

### *TOMM40* 

With longitudinal cognitive ability data, a GWAS study reported that the rs2075650 SNP in the *TOMM40* gene, which is adjacent to the *APOE* gene, was significantly associated with age-related cognitive decline (mean age: 64.6~79.1 years) (81). After fine SNP mapping of the *TOMM40*/*APOE* region, both *APOE* rs429358 and *TOMM40* rs11556505 were correlated with cognitive aging (81). Furthermore, SNPs within the *TOMM40*/*APOE* zone possessed a non-protein-coding regulatory and functional effect in a functional genomic analysis, indicating that the *TOMM40*/*APOE* zone may be linked with nonpathological cognitive aging (81). The *TOMM40* gene, located on chromosome 19q13.32, encodes the mitochondrial outer membrane complex relevant to the channel-forming subunit of the translocase, which is indispensable for construction of protein precursors to mitochondria (82).

### Other Potential Genes

The *DTNBP1* gene encodes a protein that plays a role in the biogenesis of organelle linked with lysosomes, platelet dense granules, and melanosomes (83). It has been suggested that the *DTNBP1* gene modulates general cognitive abilities both in schizophrenia patients and in healthy subjects in Japanese (mean age: 34.1~39.2) and German (mean age: 24.8) populations (84–87). In addition, Burdick et al. found that the CTCTAC risk haplotype of 6 SNPs including rs909706, rs1018381, rs2619522, rs760761, rs2619528, and rs1011313 in the *DTNBP1* gene was associated with general cognitive ability and cognitive decline in schizophrenia patients (84).

In a recent systems genetics study applying a genetically diverse population of mice, Neuner et al. pinpointed *Hp1bp3* gene to be a novel modulator of cognitive aging (88). Their findings also confirmed that as compared to cognitively healthy individuals, levels of HP1BP3 protein were significantly decreased in the hippocampi of elderly subjects with cognitive impairment, suggesting that reduced expression of *Hp1bp3* may contribute to cognitive aging in both mice and humans (88). The *HP1BP3* gene is located on chromosome 1p36.12 and encodes a histone H1 related protein with non-redundant and specific roles vital for viability and gain in humans (89).

It is worth mentioning a potential gene called *SRR* although, to our knowledge, there are only animal studies but no population studies for this gene in cognitive aging. *SRR* encodes the serine racemase enzyme which converts L-serine to d-serine. d-serine is an endogenous co-agonist for N-methyl-D-aspartate receptors (90). The *SRR* rs408067 SNP, located in the promoter region, may affect the transcription activity of the *SRR* gene (91). Reduced *SRR* expression impaired hippocampal age-related cognitive function in an animal study, suggesting that the *SRR*-dependent pathway might be one possible target of the hippocampus-related cognitive decline in aging (92). Another expression profiling study reported that various genes that are associated with cognitive ability were influenced by the *SRR* mutation (93).

## Limitations in Current Studies

Notwithstanding, there were several limitations with respect to the aforementioned studies. First, there is certainly room for development of much further research and comprehensive evaluation to reassess whether the current results remain in other ethnic populations for the investigated genetic variants with cognitive aging in terms of the association and interactions (62).

Second, given the relatively young mean age of the sample in several studies mentioned previously, the current results are unable to be extrapolated to much older populations that have higher risk for developing age-associated diseases and neurodegenerative disorders, such as MCI, AD, and other dementias (18).

Because of logistical and ethical matters, it is challenging to assess homogeneous genetic backgrounds and recruit a large enough cohort of participants at the same time (94). Furthermore, some findings were not replicated, and the discordant results found among these studies may be due to issues in the sample size, ethnicities, study design, and phenotype definitions. Moreover, confounding factors may not be fully handled, and thereby considerable bias may not be excluded.

In order to reinforce the statistical findings, it is appealing to seek more supplementary biologically relevant evidences owing to the fact that the investigated SNPs might be greatly enhanced in association studies (95).

Besides, the aforementioned studies utilized various methods to assess cognitive function. A major challenge is to ensure a proper approach for evaluating cognitive function. The well-established MMSE approach is chosen to evaluate cognitive function in several aforementioned studies because it is the most widely used screening test of cognition (96). Nevertheless, the ceiling effect of MMSE in healthy young subjects as well as its floor effect in the oldest subjects diminishes the variability (96). Similar to MMSE, another strategy is the General Practitioner Assessment of Cognition (GPCOG) with psychometric properties. However, using the GPCOG is required to further examine for its possible language or cultural tendency (97, 98). Another more recent language independent method is the CANTAB, a visual and cognitive assessment tool used on computers (99). Nonetheless, because the correlations between CANTAB and commonly used cognitive tools such as GPCOG and MMSE are only modest, the application of CANTAB should be justified in future studies (100). Moreover, an alternative is the ADAS-Cog which achieves higher sensitivity with a change of four-point (101). Although the ADAS-Cog is a well-validated scale in cognitive performance, the drawback is that about 40 min is needed to complete the task and this fact causes it unacceptable in most large-scale studies (96).

Cross-sectional design for cognitive aging studies has been adopted by many researchers because it is less feasible to examine aging trajectories for individual participants with longitudinal studies due to the high cost and long follow-up time (102). Nevertheless, it is always important to recognize the limitations of transversal studies on aging. Most importantly, we are unable to make longitudinal or causal inferences about changes in cognitive function by using cross-sectional data.

## Future Outlook

Over the past decade, advances in genome science have spawned numerous lines of research into precision medicine and multi-omics (103). In spite of spectacular progress in precision medicine and multi-omics technology, which can assemble a mammoth amount of multi-omics data, there are no established approaches to take advantage of that data in a predictive fashion (103). Therefore, we face a challenge of developing a fundamental, personalizable, mathematical model, which is calibrated on a broad range of multi-omics and clinical data (103). To conquer this challenge, a key component of future projects is to be able to advance the aforementioned predictive capability by facilitating machine learning and predictive approaches (103).

Building up a set of genetic biomarkers which are immensely dependable as a benchmark of disease status or drug response for cognitive aging will be considerably indispensable in the future (104). At this juncture, no genetic biomarkers found in the aforementioned studies would be unquestionably qualified to be incorporated in the panel owing to the aforementioned limitations (104).

Moreover, machine learning and predictive techniques such as Bayesian networks may present a conceivable approach to forecast novel drug efficacy and establish statistical models for predicting disease status (103). In future research, we will be able to help physicians in the prescription by creating predictive models which forecast the likelihood of diseases or treatment response (103, 104). In addition, predictive and machine learning approaches such as Bayesian networks might be important in weighing correlations in RNA–RNA molecule, correlations between miRNA and mRNA, as well as interactions between gene and environment (105). Moreover, the statistical modeling such as meta-analysis, pathway analysis, and gene–gene expression correlations is intrinsic to eliminate the false positive biomarkers observed during the association analyses of current precision medicine studies (105).

Essentially, evidence shows that multi-omics data and biomarkers such as genetic, epigenetic, metabolomic, transcriptomic, and proteomic profiles are important in assorted pathophysiology for a certain disease and novel drug treatment (103, 105). Subsequently, the systematic and integrative analyses of different profiles with apparently cooperative functions might have a big impact on the disclosing for the mysterious pathogenic processes of a certain disease and novel drug treatment (103, 105–107). Finally, in order to unquestionably carry out disease pathogenesis as well as novel drug therapy, future studies will have to accomplish an integrative and systematic way of using clinical information, biomarkers, and multi-omics data (103, 105).

## Summary

In this review, we have focused primarily on recent findings and relevant studies for age-related cognitive decline. The current review also highlighted the merit of association studies with relatively large sample size to incorporate a wide variety of populations for cognitive aging. In order to advance personalized treatment and prevention strategies worldwide, a main challenge is how best to integrate these findings with other pieces until the picture of cognitive aging is adequately apparent. Similarly, these findings have indicated that machine learning and predictive tools might be beneficial for clinical decision making by integrating multi-omics data and biomarkers.

In light of recent developments, novel machine learning and predictive algorithms will be the new frontier in the decades to come for establishing prognostic and diagnostic assessments by using huge data technologies for precision medicine (103, 105). Future research using machine learning and predictive approaches is warranted in the matter of managing the interactions of biomarkers and foretelling the relationship between biomarkers and drug response in precision medicine studies (103, 105). In our opinion, yet a number of challenges remain and a host of deeply key and crucial research issues must be ironed out. As we enter a period of the new envisioned science of precision medicine, personalized therapy for individuals would undoubtedly become a reality.

## Author Contributions

EL, H-YL, and C-HL involved in conception and design; EL and C-HL involved in literature review and interpretation, and manuscript writing; EL, H-YL, and C-HL involved in financial support and final approval of manuscript.

## Conflict of Interest Statement

The authors declare that the research was conducted in the absence of any commercial or financial relationships that could be construed as a potential conflict of interest.
